# Referral Practices for Cognitive Behavioral Therapy for Insomnia: A Survey Study

**DOI:** 10.1155/2015/819402

**Published:** 2015-07-21

**Authors:** Deirdre A. Conroy, Matthew R. Ebben

**Affiliations:** ^1^Department of Psychiatry, University of Michigan Hospitals and Health Systems, Ann Arbor, MI 48109, USA; ^2^Center for Sleep Medicine, Weill Medical College, Cornell University, New York, NY 10065, USA

## Abstract

This study examined referring practices for cognitive behavioral therapy for insomnia (CBTI) by physicians at University of Michigan Hospitals and Weill Cornell Medical College of Cornell University. A five-item questionnaire was sent via email that inquired about the physician's patient load, number of patients complaining of insomnia, percent referred for CBTI, and impressions of what is the most effective method for improving sleep quality in their patients with insomnia. The questionnaire was completed by 239 physicians. More physicians believed a treatment other than CBTI and/or medication was most effective (*N* = 83). “Sleep hygiene” was recommended by a third of the sample. The smallest number of physicians felt that CBTI alone was the most effective treatment (*N* = 22). Additional physician education is needed.

## 1. Introduction

Insomnia is one of the most common health complaints. Population prevalence estimates vary between 9% [1] and 57% in older individuals [2]. In the recent past, chronic insomnia was almost always viewed as a symptom of medical and mental disorders [3]. In this conceptualization, it made sense to focus on the evaluation and treatment of the underlying causes of trouble sleeping. The new conceptualization of insomnia, as codified in the DSM V [4] and ICSD 3 [5], acknowledges insomnia's independent status as a condition warranting clinical attention. The implications of this new view are that clinicians should not wait until they have addressed the medical and psychiatric comorbidities before they intervene in sleep disturbance.

The recent evidence of the effects of insomnia on medical and mental disorders has helped establish the reciprocal or bidirectional relationship between insomnia and medical and mental disorders [6–9]. Furthermore, the development of effective nonpharmacological treatments, such as cognitive behavioral treatment for insomnia (CBTI), that demonstrates effectiveness in insomnia with and without a wide range of comorbidities has given physicians and sleep specialists a larger set of therapeutic options compared to what were available in the past [10–13]. Taken together, the understanding that insomnia is not just a symptom of other disorders, nonpharmacological treatments are as effective as medications [14], and insomnia is common predicts that physicians can be expected to rely more on specialists who are adept at treating sleep disturbance. However, there is a mismatch between the number of patients with insomnia and those receiving CBTI treatment. The issue of access to clinicians with expertise in delivering this therapy has been raised as one of the reasons accounting for this mismatch [15]. However, even in clinical practices where these services are available, clinicians with CBTI training may not experience the high patient volume one might expect. This may be due, in part, to patients' preference for a quick fix for insomnia with medications [16]. Another hypothesis is that physicians may not be aware of CBTI as a viable treatment option for their patients or they may not be knowledgeable about the therapy, despite its widespread use. Moreover, two recent studies highlight the importance of using CBTI as the primary tool to treat insomnia [17, 18]. Both studies found an increase in mortality associated with the use of hypnotic medication.

The purpose of this study was to investigate the referral practices and attitudes of physicians towards insomnia treatment at two academic medical centers in the United States. We sought to examine physician's opinions of treatment efficacy of medication and/or CBTI for the treatment of insomnia. This information may be useful in determining a strategy to educate medical providers on effective nonpharmacological treatment options for patients with insomnia.

## 2. Methods

### 2.1. Participants

In order to obtain a diverse sample, we selected two large area hospitals in the Midwest and Eastern United States. Weill Cornell Medical Center is part of New York-Presbyterian University Hospital of Columbia and Cornell and is located in Manhattan, NY. It is a 2,264-bed general medical and surgical facility with 105,339 admissions in the most recent year reported. It performed 30,739 annual inpatient and 76,689 outpatient surgeries. New York-Presbyterian University Hospital of Columbia and Cornell is a teaching hospital. Weill Cornell Medical Center has two clinical psychologists in faculty at the Center for Sleep Medicine that are certified in behavioral sleep medicine. The University of Michigan Hospitals and Health Centers is located in Ann Arbor, MI. It is a 919-bed general medical and surgical facility with 45,137 admissions in the most recent year reported. It performed 17,784 annual inpatient and 28,270 outpatient surgeries [19]. It is also a teaching hospital. There are three clinical psychologists in faculty in the Behavioral Sleep Medicine Clinic at the University of Michigan Hospitals and Health Systems that are certified in behavioral sleep medicine.

### 2.2. Questionnaire Survey

A five-item questionnaire was created by the authors to illicit information about clinical specialty, patient flow, and referral practices for CBTI. The items included the following. (1) What is your primary specialty? (2) What is the total number of patients you see per month? (3) How many patients do you see per month that complain of insomnia? (4) What percentage of your patients do you refer for cognitive behavioral therapy for insomnia (CBTI)? (5) What treatment do you find is the most effective for improving sleep quality in patients with insomnia: (A) medication, (B) CBTI, (C) a combination of CBTI and medication, and (D) other? A text response could then be entered. The questionnaire was conducted with Survey Monkey, internet based survey software.

An email with the link to the survey was sent through an email that reached the medical staff at the University of Michigan Hospitals and Health Systems (*n* = 1600) and New York Presbyterian Hospital-Cornell Medical Center (*n* = 1511). Respondents completed the survey by clicking a link to the survey website included in the email. The project was exempted by both the University of Michigan (HUM00079350) and Weill Cornell Medical College (1308014173) Institutional Review Boards.

## 3. Data Analysis

Quantitative data was analyzed using SPSS version 22 (IBM Software, Armonk, NY). A one-sample chi-square test was conducted to assess differences in proportions. Equal frequencies were expected among responses to questions. Qualitative analysis was used to evaluate free text responses. This was performed with the Text Analysis tool provided by Survey Monkey. All terms yielding response rates greater than 10% are described in Results. Terms used in less than 10% of responses are listed only if they could have been viewed as belonging to the “medication” or “CBTI” answers to question 5 of the survey.

## 4. Results

A total of 239 clinicians responded to the survey. Most respondents considered themselves either internal/family medicine physicians (*n* = 57, 24.3%) or “other” (*n* = 50, 21.3%). Other common specialties included pediatrics (*n* = 28, 11.9%), psychiatry (*n* = 17, 7.2%), surgery (*n* = 14, 6.0%), and oncology (*n* = 11, 4.7%). The remaining specialties included pulmonology (2.1%), nursing (0.9%), ENT (2.6%), cardiology (3.0%), psychology (3.4%), neurology (2.6%), urology (0.9%), OB/GYN (3.0%), GI (0.9%), sleep medicine (1.7%), or geriatrics (3.8%). See Table 1 for a breakdown of prescribing practices by specialty.

Respondents saw an average of 121 (SD = 106) patients per month (range 0–1000) and an average of 15.2 (SD = 21) patients per month with insomnia (~12% patients with insomnia per month). However, the average number of patients referred for CBTI per month was 1.5 (SD = 4.9). Opinion of treatment efficacy was significantly different among responders to the survey,* X* ^2^ (3, *N* = 213) = 35.02, *p* = 0.000. The effect size was 0.06.

**Figure 1:**
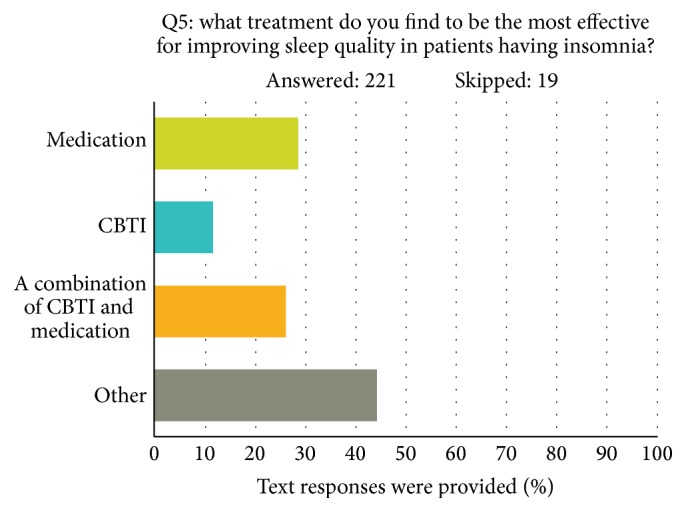


The number of physicians who believed a treatment other than CBTI and/or medication was most effective (*N* = 83) was much larger than the hypothesized number of 53.3 (see Figure 1). A qualitative analysis of these responses revealed that 29.6% contained the term “sleep hygiene,” and 4% used the term “relaxation.” The word “medication” or “melatonin” was used in 7.1% of responses. Other commonly used terms included “no experience” (18.4%), “treat medical condition” (12.2%), “not applicable” (11.2%), and “refer to PCP” (11.2%). The smallest number of physicians felt that CBTI alone was the most effective treatment (*N* = 22). A total of *n* = 55 and *n* = 53 doctors thought that medication and a combination of medication and CBTI, respectively, were the most effective treatment approaches. Other text responses to other treatments included remedies such as “warm milk and hydroxyzine” as treatment for insomnia.

## 5. Discussion

The purpose of this study was to investigate the treatment approaches used for insomnia by medical professionals at two large academic medical centers in the United States. The study shows that although respondents in our survey see many patients per month, very few (approximately 1%) of these patients are referred for treatment with CBTI alone. This is particularly surprising given that both the University of Michigan and Weill Cornell Medical College have well known sleep laboratories with practitioners certified in behavioral treatments for insomnia. Therefore, the dearth of referrals could not be attributed to a lack of appropriate treatment facilities, which could be the case elsewhere. The responses to “other” for question number 5 “What treatment do you find is the most effective for improving sleep quality in patients with insomnia?” suggest that the term CBTI may not be well understood. Physicians commonly reported recommending “sleep hygiene” and “relaxation techniques” instead of CBTI, even though these are components of the multimodal CBTI approach to treatment. This is consistent with a similar large survey study of 296 general practitioners in England, which revealed that sleep hygiene advice is provided by 88% of GPs [20]. Moreover, recommending components of CBTI, such as “sleep hygiene” alone, which have not been shown to independently improve sleep quality [21], may lead to poor patient treatment outcomes. This study suggests that although scientifically rigorous research studies have found CBTI effective for insomnia and numerous disorders comorbid with insomnia [22], additional education to the medical community on CBTI is needed. Educational programs may include but are not limited to hospital seminars, grand round lectures, colloquiums, or mentorships of residents or other health care professionals in CBTI. Training programs for nonsleep specialists [23], such as the one utilized by the Veterans Health Administration, provide an example of an effective way to train clinicians in CBTI [24] and improve insomnia in that patient population [25].

One limitation to our study is that the University of Michigan and Weill Cornell Medical College/New York-Presbyterian Hospitals are tertiary referral hospitals with few primary care doctors. Therefore, other regional medical centers, with a higher overall percentage of primary care practitioners, may have different referral patterns. However, most regional centers do not have behavioral sleep medicine experts, and many of the referrals made to sleep laboratories (most behavioral sleep medicine specialists are affiliated with a sleep laboratory) are made by pulmonologists, ear, nose, and throat specialists, and cardiologists. As a result, we believe that our data provide a fair analysis of the referral network seen by many behavioral sleep medicine experts.

## 6. Summary

CBTI is the first line treatment for insomnia and is now well validated as being effective in the short and long term [26]. A brief survey completed by 239 medical professionals at two large academic medical centers revealed that therapies other than CBTI seem to be most commonly recommended for patients with insomnia. Medical education programs within medical settings to promote proper insomnia treatments are needed.

## Figures and Tables

**Table 1:** Referral practices for insomnia treatment and patient volume by physician specialty.

	What treatment do you find to be the most effective for improving sleep quality in patients having insomnia?				What is the total number of patients you see per month?		How many patients do you see per month that complain of insomnia?	
	Medication	CBTI	CBTI and medication	Other	Mean	Standard deviation	Mean	Standard deviation
	Count	Count	Count	Count				
Specialty								
GP	20_a,b_	8_a,b_	25_a_	18_b_	144	93	20	21
Specialist	29_a_	7_a_	26_a_	43_a_	118	117	16	23
Pediatrics	2_a_	7_b_	0^1^	15_b_	110	95	6	12
Surgery	4_a_	0^1^	2_a_	6_a_	89	77	6	11

Note: values in the same row and subtable not sharing the same subscript are significantly different at *p* < 0.05 in the two-sided test of equality for column proportions. Cells with no subscript are not included in the test. Tests assume equal variances.^2^

^1^This category is not used in comparisons because its column proportion is equal to zero or one.

^2^Tests are adjusted for all pairwise comparisons within a row of each innermost subtable using the Bonferroni correction.
